# Formation of Self-Assembled Mesophases During Lipid Digestion

**DOI:** 10.3389/fcell.2021.657886

**Published:** 2021-06-11

**Authors:** Anna C. Pham, Andrew J. Clulow, Ben J. Boyd

**Affiliations:** ^1^Drug Delivery, Disposition and Dynamics, Monash Institute of Pharmaceutical Sciences, Parkville, VIC, Australia; ^2^ARC Centre of Excellence in Convergent Bio-Nano Science and Technology, Monash Institute of Pharmaceutical Sciences, Parkville, VIC, Australia

**Keywords:** lipid, liquid crystalline, self-assembly, digestion, lipolysis, mesophase

## Abstract

Lipids play an important role in regulating bodily functions and providing a source of energy. Lipids enter the body primarily in the form of triglycerides in our diet. The gastrointestinal digestion of certain types of lipids has been shown to promote the self-assembly of lipid digestion products into highly ordered colloidal structures. The formation of these ordered colloidal structures, which often possess well-recognized liquid crystalline morphologies (or “mesophases”), is currently understood to impact the way nutrients are transported in the gut and absorbed. The formation of these liquid crystalline structures has also been of interest within the field of drug delivery, as it enables the encapsulation or solubilization of poorly water-soluble drugs in the aqueous environment of the gut enabling a means of absorption. This review summarizes the evidence for structure formation during the digestion of different lipid systems associated with foods, the techniques used to characterize them and provides areas of focus for advancing our understanding of this emerging field.

## Introduction

Dietary lipids are essential for cellular function and for providing and storing energy in the body. Lipids including triglycerides, phospholipids, and cholesterol are present in a wide variety of foods including fish, vegetables, eggs, meat, nuts, and dairy products. Over 98% of lipids are absorbed by the body following digestion and serve various biological roles (Carey et al., 1983). In infants, the consumption of lipids from human breast milk or infant formula provides approximately 45–55% of dietary energy intake (Castillo and Uauy, 2003). The absorption of long-chain polyunsaturated fatty acids (LCPUFA) such as arachidonic acid and docosahexaenoic acid from infant formula has been shown to be beneficial for cognitive development in infants (Heird, 2001). Furthermore, the metabolism of arachidonic acid produces eicosanoids, which are signaling molecules involved in various anti-inflammatory responses (Lone and Taskén, 2013). Other types of lipids found in human breast milk or infant formula are also essential for growth, improved visual function, and the development of the immune system (Friel and Qasem, 2016). Following infancy, the consumption of dietary lipids continues to provide a rich source of energy for humans (Sikorski et al., 2010). Absorption of fat during digestion not only regulates satiety and energy stores but also assists with the uptake of lipid-soluble vitamins (Goncalves et al., 2015). Other roles that lipids serve in the body include the maintenance of the structural integrity of cells, prevention of water loss in cells, and providing precursors for the synthesis of essential metabolites (Sikorski et al., 2010).

Lipids are not only an essential dietary component but are also often used to facilitate the oral delivery of poorly water-soluble drugs. Nearly 40% of new drug entities are poorly water-soluble, which means that whilst they may exhibit high membrane permeability they suffer from poor dissolution in the aqueous gastrointestinal environment, which hinders their bioavailability (Lipinski, 2002). Co-administering poorly water-soluble drugs with lipids is a useful strategy for improving bioavailability by avoiding the need for dissolution in the gastrointestinal tract and taking advantage of the lipid digestion pathway (Humberstone and Charman, 1997). Lipid absorption, and the use of lipids to dissolve and absorb poorly water-soluble nutrients and drugs critically relies on digestion of the lipids and the subsequent interaction of the co-administered drugs/nutrients with the lipid digestion products (Boyd et al., 2018).

## The Process of Digestion of Dietary Lipids

**FIGURE 1:**
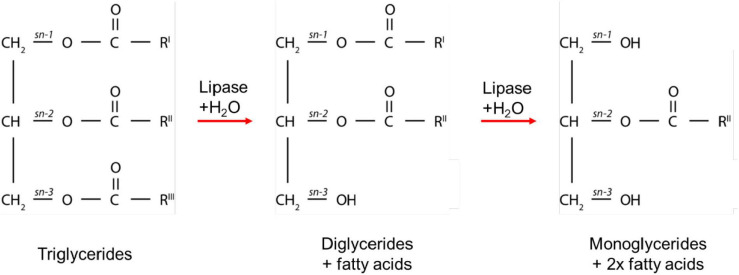
A schematic diagram of the digestion process of triglycerides. R^*I*^, R^*II*^, and R^*III*^ represent the alkyl chains that make up the triglycerides, which can comprise the same or different fatty acids. The combinations of alkyl chains are dependent on the source of fat.

The enzymatic digestion of lipids begins in the mouth with lingual lipase partially digesting triglycerides to form diglycerides and fatty acids (Teresa et al., 1984). The digestion continues in the gastric compartment where gastric lipase is secreted from chief cells lining the gastric mucosa. Approximately 10–30% of lipids are hydrolyzed in the stomach by gastric lipases to form a crude emulsion containing diglycerides, monoglycerides, and fatty acids (Carey et al., 1983; Armand et al., 1996). The mechanical churning of the stomach and rhythmic contractions several times per minute act to reduce the particle size of the food chyme to less than 0.5 mm in diameter so that it is able to pass through into the duodenum (Meyer et al., 1976). Triglycerides and diglycerides are then further digested by pancreatic lipase to form two moles of fatty acid and one mole of monoglyceride for each mole of triglyceride originally consumed (Armand et al., 1996; Figure 1). The acidic content entering the duodenum from the stomach triggers bicarbonate secretion to increase the pH to approximately 6.2 to 8.1 to allow for optimal lipase activity (Embleton and Pouton, 1997; Kalantzi et al., 2006). Monoglycerides and free fatty acids entering the duodenum also signal for the secretion of bile salts, phospholipids, and cholesterol from the gall bladder to act as an endogenous surfactant, which coats and stabilizes emulsion droplets (Borgström and Patton, 1991). This enables water-soluble colipase/lipase complexes to act at the oil-water interface of the emulsion droplets to hydrolyze fatty acids from the triglycerides, diglycerides, and possibly monoglycerides (Persson et al., 2007). Triglycerides are typically stereospecifically hydrolyzed initially at the sn-3 position, followed by the sn-1 position to form 2-monoglycerides and fatty acids (Borgström and Patton, 1991). These products of digestion then self-assemble into mixed colloidal structures such as vesicles, mixed micelles, and liquid crystalline mesophase systems (Salentinig et al., 2013; Phan et al., 2014).

The pathway by which the fatty acids are transported after absorption is dependent on the chain-length (Hamilton and Kamp, 1999). Digestion to form short to medium fatty acids and monoglycerides results in the passive diffusion of the digestion products across the intestinal epithelium into the enterocytes for direct transport to the portal vein (Porter et al., 2007). Long-chain fatty acids and monoglycerides that have been absorbed into the enterocytes are transported into the endoplasmic reticulum to be re-esterified into triglycerides. The triglycerides are incorporated with cholesterol, proteins, and phospholipids to form lipoproteins called chylomicrons (Stremmel et al., 2001). Lipoproteins are taken up by the lymphatic system and directly enter the systemic circulation, bypassing first pass metabolism in the liver (Stremmel et al., 2001). While these processes post-absorption do not relate directly to the formation of ordered lipids mesophases in the gastrointestinal tract, the rate of availability of the lipids and other components as a consequence of structure formation is likely important for these processes but the links have not yet been made.

## Types of Lipid Mesophases

### Lipid Self-Assembly and the Critical Packing Parameter Concept

The amphiphilic lipids formed upon digestion of triglycerides can self-assemble into ordered structures in the intestinal fluids. The lipids generated from dietary triglycerides are primarily unsaturated and saturated medium chain or long-chain amphiphilic molecules (Pham et al., 2020). Upon liberation by lipase and exposure to excess water, the hydrophilic head groups are specifically hydrated, whilst the lipophilic chains associate with each other to avoid interaction with water. The packing geometry of the lipids results in their self-assembly into different mesophases, with common mesophases observed being of the “inverse” or type 2 topology where the curvature of the interface is toward the aqueous domains. These mesophases include the disordered inverse micellar (L_2_), inverse bicontinuous cubic (V_2_), inverse hexagonal (H_2_) phase, and inverse micellar cubic (I_2_, Fd3m space group) phase as well as the fluid lamellar (L_α_) phase. The V_2_ phase can be further divided into space groups: diamond (Pn3m), gyroid (Ia3d), and primitive (Im3m) V_2_ phases. Particles with the L_α_, V_2_, and H_2_ mesophases constituting the internal structure are termed liposomes (or vesicles), cubosomes, and hexosomes, respectively. The propensity for different structures to be formed is dependent on the dynamic lipid composition and conditions such as pH and ionic strength. Without preempting too much of discussion to come, it is then conceivable that as droplets of lipids from food form these structures during digestion that the droplets would possess such structures and therefore transform from essentially unstructured triglyceride droplets into cubosomes or other structures depending on the local lipid packing inside the droplet.

The concept that relates the self-assembly of lipids into ordered mesophase structures to the geometry of lipid packing is known as the critical packing parameter (CPP). The value of the CPP is calculated by the ratio of the hydrophobic tail volume of the amphiphilic molecule to the product of the effective head group area and the length of the surfactant tail (Eq. 1) (Israelachvili et al., 1976).

**FIGURE 2:**
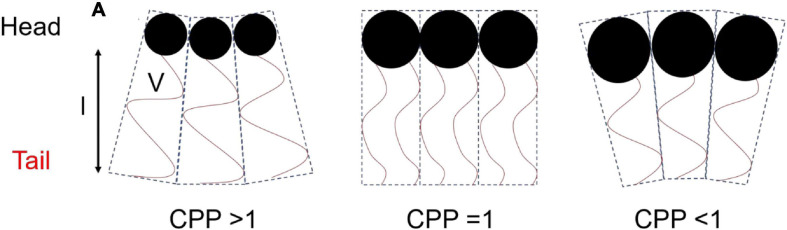
Schematic diagram of the geometry of packing of amphiphilic molecules and corresponding CPP values. This image was based on and redrawn from Du et al. (2014).

(1) Equation 1:CPP=Val

where *V* is the volume of the hydrophilic tail, *a* is the effective area of the head group, and *l* is the length of the surfactant tail (Figure 2). Molecules with larger head groups and smaller hydrophobic chains tend to pack to form type 1 structures which favors curvature toward the lipophilic region of the mesophase (positive curvature). As mentioned above the type 2 structures or inverse structures are formed when curvature is toward the hydrophilic region of the mesophase (negative curvature) and are more commonly found in biological settings and pharmaceutical research (Seddon et al., 2000; Shearman et al., 2006).

The CPP is affected by the temperature of the surrounding environment, ionic strength, additives, and pH of the molecule if it is ionizable (Czeslik et al., 1995; Liu et al., 2013; Tangso et al., 2013; Salentinig et al., 2014). For example, an increase in temperature would effectively increase chain movement which increases *V*, leading to an increased CPP and curvature toward the aqueous compartment (Israelachvili, 2011).

## Techniques for Studying Lipid-Based Mesophase Systems During Digestion

### *In vitro* Digestion Models

**FIGURE 3:**
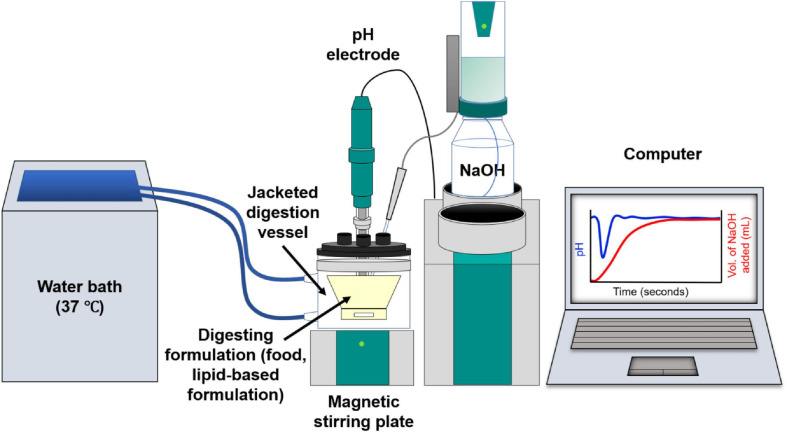
Schematic diagram of the *in vitro* digestion pH-stat apparatus. Digestion of lipids from the formulation generates fatty acids resulting in a drop in pH. This is compensated by automated addition of NaOH to maintain a fixed pH value, enabling the tracking of digestion kinetics on the computer.

*In vitro* digestion models enable lipid digestion to be conducted under controlled simulated human gastrointestinal conditions. *In vitro* digestion studies are used to obtain information on the digestion kinetics of a lipid system, the extent of digestion over a given time, and in pharmaceutical applications, the drug distribution within the lipid and aqueous phases (Porter et al., 2004). The most commonly used *in vitro* lipolysis model is the pH-stat model (Figure 3; Dahan and Hoffman, 2006, 2008; Fatouros et al., 2007). Using the pH-stat model, the pH is maintained at a specific pH value representative of *in vivo* conditions during digestion, typically at a value between pH 6.5 and 7.5 representative of the small intestine. As the triglycerides are digested by lipase, the fatty acids that are liberated act to reduce the pH of the medium, and the pH-stat system will titrate against those fatty acids through addition of NaOH to maintain the pH at its set point. The amount of NaOH consumed during the digestion is measured over time, enabling a kinetic digestion profile to be established. Samples can be taken during the digestion process and analytical techniques such as gas chromatography or high performance liquid chromatography (HPLC) with mass spectrometric detection can be used to determine the lipid species present (Pham et al., 2020), the degree of drug solubilization or drug precipitation upon digestion of particular lipid systems (Anby et al., 2014; Khan et al., 2015a,b). Other *in vitro* digestion models also include gastric compartments to encompass more of the lipolysis processes occurring *in vivo* (Lee et al., 2013; Ye et al., 2016; Berthelsen et al., 2019).

Lipase inhibitors such as 4-bromophenylboronic acid (4-BPBA) or orlistat can be used to halt the digestion to enable offline analysis of the liquid crystalline (LC) mesophases that form during the lipolysis process. However, the addition of the inhibitor introduces an element of uncertainty as to the effect of the inhibitor on mesophase formation and 4-BPBA was shown to disrupt the packing of some mesophase which would otherwise be present during “online” *in vitro* digestion (Phan et al., 2013). There is also the treatment and storage of samples taken from a digestion for inhibition prior to study by an appropriate analytical technique that also make “offline” structural determination unattractive. Ideally *in situ* determination of mesophase formation during digestion would be utilized to circumvent these issues, a development described later.

A disadvantage of using the aforementioned *in vitro* lipolysis methods is that there is no absorptive sink present to remove lipid digestion products that would otherwise occur through absorption *in vivo*. The rate of digestion is likely to be faster than absorption (and must precede it in any case) so at least some accumulation of lipid digestion products is to be expected *in vivo* during digestion. Nevertheless, to attempt to address the limitation of the pH-stat model, an absorptive compartment can be included where two digestion vessel compartments are separated by a monolayer of Caco-2 cells (Keemink and Bergström, 2018). Caco-2 cells are similar to the human epithelial cells of the intestine (Sambuy et al., 2005). However, this system requires optimization to prolong the integrity of the Caco-2 cells that are often damaged by the presence of pancreatic lipase or the digestion media (Bu et al., 2016; Sadhukha et al., 2018). Recently, the Caco-2 cells in combination with HT-29-MTX cells (human colon cell line) have been proposed as an alternative absorptive sink for *in vitro* digestions (Hempt et al., 2020). The addition of the HT-29-MTX cells provides a protective layer to the cell monolayer attributed to the mucus secretion from the HT-29-MTX cells. Though cell viability was improved, the evolution of LC structures during the digestion of bovine milk with and without the added Caco-2/HT-29- MTX cells was similar to that with no cell sink, which suggested that more optimization is required to incorporate a fully functional absorptive sink.

*In vitro* digestion models only provide information on digestion kinetics and other techniques must be employed either on samples retrieved over time or *in situ* to qualitatively and quantitatively analyze the presence of lipid mesophases within a system. Earlier studies have used microscopy to visualize the process of digestion, in which the discovery of the L_α_ and “viscous isotropic” phases were observed during the digestion of olive oil under simulated physiological conditions (Patton and Carey, 1979). More recently, coupling *in vitro* digestion models to advanced techniques such as small angle X-ray scattering (SAXS) and small angle neutron scattering (SANS) can now provide in-depth information on the structural characterization on a nanoscale (Hyde, 2001; Kirby et al., 2013) as described further below.

### Microscopy Techniques

#### Light Microscopy

**FIGURE 4:**
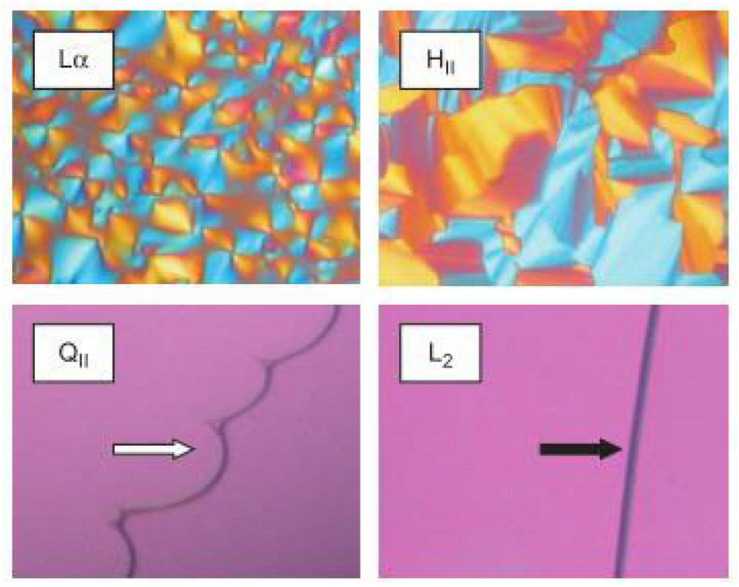
Images of various LC mesophases captured using CPLM. The upper panels are examples of CPLM images of anisotropic mesophases like L_α_ and H_2_ phases show birefringence. The dark appearances on the bottom panels are due to the isotropic nature of the V_2_ (called Q_*II*_ in the original work this image is reproduced from) and L_2_ phases. The V_2_ and L_2_ phases can be distinguished by their appearance at the lipid/water interface. Images were reproduced with permission from Boyd et al. (2009).

Crossed polarized light microscopy (CPLM) is a simple technique that can be used to rapidly characterize the internal structure of a bulk material during digestion (Patton and Carey, 1979; Dong et al., 2006). The bulk material, that is the lipid that has been equilibrated in excess water, is assumed to behave in the same manner as its nano-sized dispersed forms which are more commonly used in oral delivery studies. The samples are generally placed between glass slides that are then viewed under crossed-polarized filters. Samples that are isotropic such as inverse micellar (L_2_) or cubic (V_2_) phases will have no light passing through the second filter which gives the images a dark appearance (Rosevear, 1954, 1968). Samples containing isotropic materials such as the L_2_ and V_2_ phase can be distinguished by the appearance at the lipid-water interface (Figure 4). The high viscosity of the cubic phase will appear structured at the lipid-water interface, while the micellar (L_2_) phase will appear flat (Rosevear, 1968). Samples that are anisotropic such as L_α_ or H_2_ phases will appear bright as they rotate plane polarized light, allowing some light to bypass both polarizer films. Lamellar phases will give a mosaic-like or more disordered brightness while hexagonal phase will give a more fan-like appearance (Rosevear, 1968). This technique is generally used as a preliminary step for characterizing mesophases during digestion as the pH, temperature, and addition of enzymes can be controlled to simulate the digestion of lipids in the gastrointestinal tract.

Confocal laser scanning microscopy is commonly used to study the localized components within cells or other types of specimens (Matsumoto and Hale, 1993). This is done by fluorescently labeling regions of interest to enable contrast from the background using fluorescent lipophilic probes such as Nile Red. Using CLSM, high resolution images can be achieved by focusing a laser onto a fixed spot within a sample, at a defined scanning depth within the sample (Matsumoto and Hale, 1993; Wu and Bruchez, 2004). Fluorescence is emitted from this defined spot and is refined by pinholes before reaching a light detector. Signals that are out of focus are removed by the pinholes to allow only the illuminated in-focus spot to be detected (Zhou and Li, 2015). To visualize mesophases, the scan differential interference contrast (DIC) mode is used obtain contrast within unstained samples (Gallier et al., 2013a,b). The sample requires no preparation and the technique is non-invasive. Information regarding the size of the lipid droplets and the structural changes on the surface of the droplets during digestion can be obtained. Confocal laser scanning microscopy is particularly useful for examining the intracellular interaction of mesophase nanoparticles and has been used to visually observe lipid droplets enveloped by lamellar phases from digested bovine milk (Zeng et al., 2012; Gallier et al., 2013a,b).

#### Electron Microscopy

**FIGURE 5:**
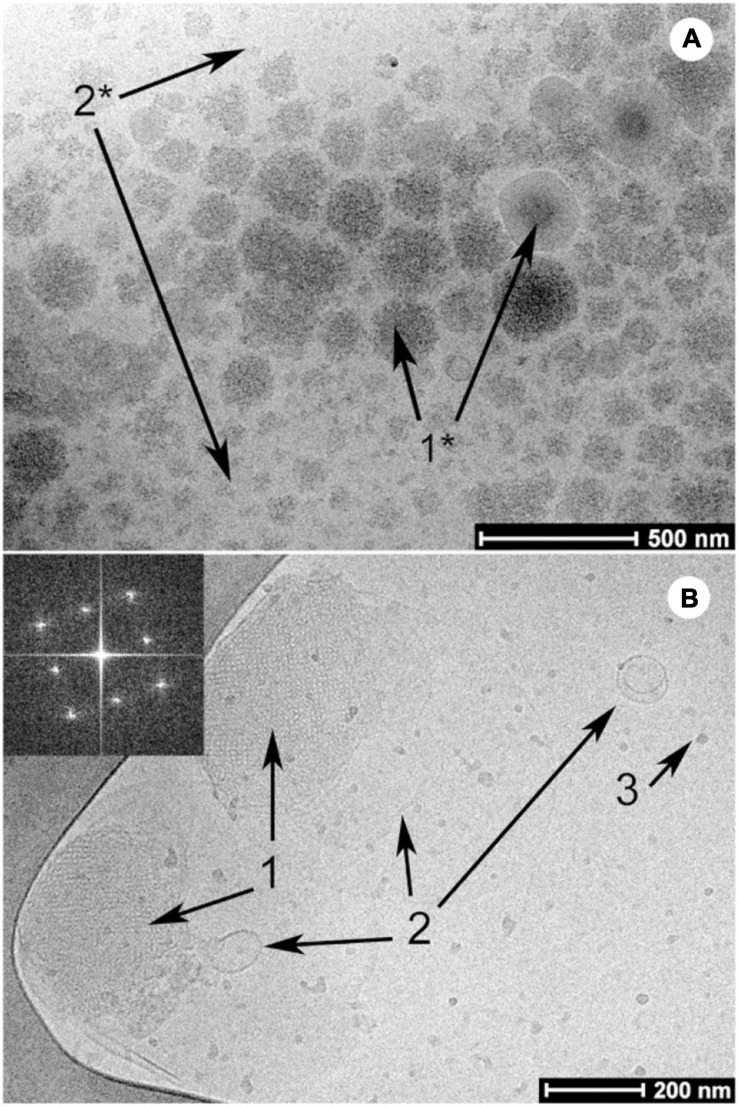
Examples of cryo-TEM images of milk before **(A)** and after digestion **(B)** by Salentinig et al., reproduced with permission from Salentinig et al. (2013). Copyright (2013) American Chemical Society. Panel **A** shows the presence of unstructured emulsion droplets characteristic of bovine milk. The presence of bicontinuous cubic phase (annotated with “1”), lamellar vesicles (annotated with “2”) and protein (annotated with “3”) can be observed in the digested milk in panel **B**.

Cryogenic-transmission electron microscopy (cryo-TEM) is another microscopy technique that can be used to study the structural morphology of lipid mesophases. The general size of the particles and their internal structure can be observed on a local scale with resolutions down to 1.8 Å (Tan et al., 2008; Fong et al., 2012; Merk et al., 2016). This technique involves pipetting the dispersed material onto a copper grid perforated with carbon films that have been prepared under glow discharge in nitrogen. The copper grid is then immediately submerged into liquid nitrogen or liquid ethane to immediately freeze the sample onto the grid. The samples are then kept under liquid nitrogen until microscopy analysis (Almgren et al., 2000; Spicer et al., 2001; Martiel et al., 2014). This enables the structural integrity of the LC mesophases to remain unaltered at the time of analysis and makes it possible to analyze mesophases in solution. The mesophases observed under cryo-TEM can be quantitatively analyzed using fast Fourier transform analysis (Sagalowicz et al., 2006). Contrary to CPLM or CLSM, the preparation procedure of cryo-TEM is labor intensive and time costly. Furthermore, samples containing high concentrations of sugars such as milk can reduce the contrast in cryo-TEM images, hence, reducing the resolution of self-assembled structures from the background (Duzgunes, 2003). Nonetheless, cryo-TEM is an effective technique for providing 3D visualization of the various mesophases that can occur during digestion (Figure 5; Phan et al., 2013; Salentinig et al., 2013).

### Scattering Techniques

**FIGURE 6:**
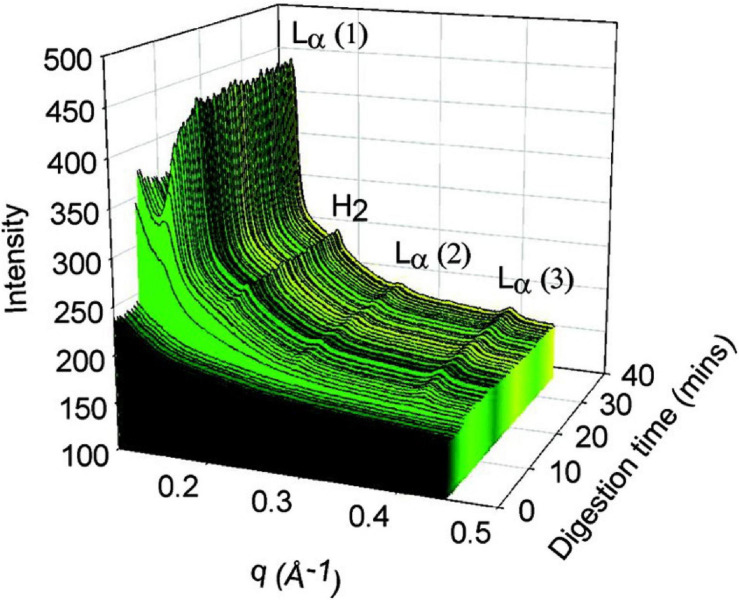
The formation of liquid crystalline structures during the digestion of a self-nanoemulsifying drug delivery system (SNEDDS) by Warren et al. (2011). Scattering profiles of the digest are shown as a function of time (min), and the peaks that arise during digestion were attributed at the time to L_α_ and H_2_ phases although it is likely from more recent studies that the lamellar phase is due to calcium soap formation (Clulow et al., 2018). Adapted with permission from Warren et al. (2011). Copyright (2011) American Chemical Society.

Small angle X-ray scattering has emerged as one of the most powerful tools for determining mesophase structure formation in lipid systems during digestion. SAXS is a non-invasive technique that involves scattering of X-rays based on the spatial variations in electron density between the aqueous and lipid regions of mesophases (Cullity, 1956; Gregory, 1957; Hyde, 2001). Ordered (crystalline or liquid crystalline) structures produce unique scattering profiles which enable identification. Some of the advantages of using SAXS are that the sample preparation is relatively simple, the acquisition time is rapid (enabling dynamic flow through measurements when using synchrotron sources), the temperature can be controlled, and highly detailed information regarding structure can be determined (Kirby et al., 2013). The real time formation of highly ordered, mesophase structures during digestion was demonstrated when the pH stat digestion model was used in conjunction with SAXS (Fatouros et al., 2007; Warren et al., 2011). An example of LC transformation during *in vitro* digestion coupled with SAXS is shown below in Figure 6.

Small angle neutron scattering is analogous to SAXS but involves scattering neutrons through interactions with atomic nuclei. The use of isotopic substitution, particularly substitution of deuterium for hydrogen in either the self-assembling molecules or the solvent can enable determination of localization of specific components in the mixture (Lopez-Rubio and Gilbert, 2009). Where samples (mesophases) have similar electron densities as their surrounding environment (solvent), SANS with deuterium labeling is used in place of SAXS to improve scattering contrast between the two (Chu and Liu, 2000). Deuterium is a heavier isotope of hydrogen, which produces small changes to the sample but significant effect on neutron scattering profiles (Ramsay et al., 2007). Deuterium substantially boosts the coherent neutron scattering power (scattering length density) of a molecule, where hydrogen reduces it. If the scattering length densities of the sample and matrix are similar or the same, there is little scattering contrast and weak or no scattering profiles are observed. By deuterating part of the sample or the matrix, the scattering length densities of the components in the system become substantially different, enhancing scattering contrast and highlighting the components of the sample that have substantially different scattering power to the matrix. The localization of components in mixed bile salt micelles from *in vitro* lipolysis as well as their shape and size can be determined using SANS (Phan et al., 2015; Rezhdo et al., 2017). In the context of studying mesophase formation during digestion, the disadvantage of using SANS is the generally lower flux and lower detector sensitivity of neutron sources than synchrotron X-ray sources, which results in long acquisition times. This precludes true “real time” *in situ* studies of changes in self-assembled structures with time resolution of shorter than several minutes per timepoint using neutrons, compared to seconds with synchrotron-based X-ray approaches.

## Formation of Mesophases During the Digestion of Lipid Systems

**FIGURE 7:**
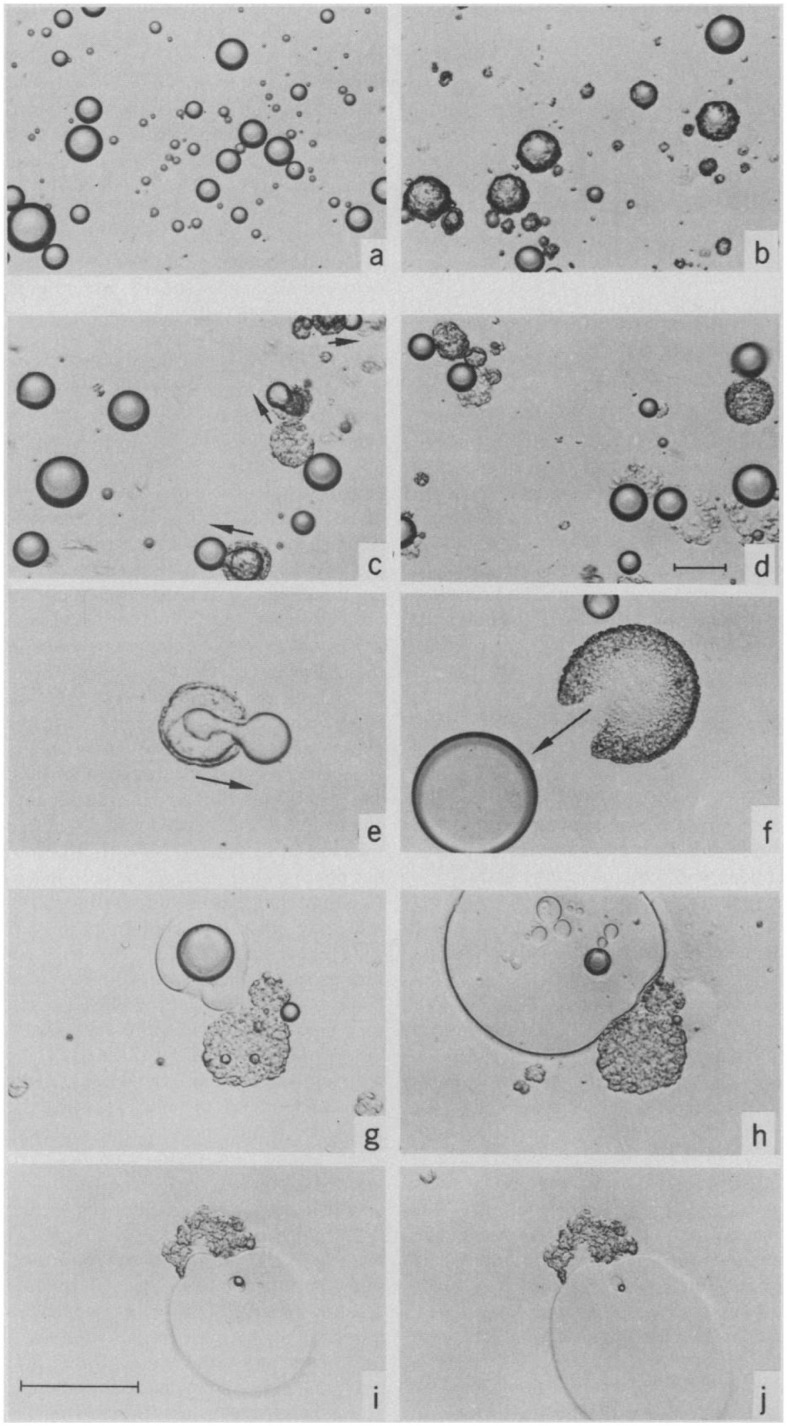
Light microscopy images capturing the digestion process of a droplet of olive oil (Patton and Carey, 1979). Reprinted with permission from AAAS. A lamellar shell was formed following initiation of digestion **(a,b)**, where the shell was later broken leading to extrusion of the undigested lipids **(c–f)**. The presence of “viscous isotrope” phases was later observed **(g–j)**.

The process of digestion is essential for the conversion of many lipid systems into mesophases. Depending on the ratio of undigested lipids to digestion products, the lipid mesophases will change as digestion progresses. As mentioned above, the changes in the microstructure of the lipids during the digestion of olive oil was visualized by Patton and Carey (1979) using light microscopy, where the formation of LC phases (L_α_ or “viscous isotrope”) was indicated (Figure 7). The digestion of triglycerides was further explored later, again using light microscopy, in which intermediate lipid mesophases were observed during the digestion of either olive oil or emulsified triolein (Patton et al., 1985). This study examined the interplay between lipase and the digesting lipid in the aqueous environment similar to that of intestinal fluids. The concept of light microscopy was explored to visually inspect the enzymatic activity of lipase on lipids that cannot be determined by simply conducting chemical reactions. The relevant digestion components were also isolated as opposed to using human intestinal aspirates to enable key interactions during digestion to be established more easily. This was because human intestinal aspirates may not be homogeneously mixed and digested, combined with the presence of other endogenous secretions that can make it more difficult to visualize the changes in the microstructure of lipids under light microscopy. A correlation between the LC structures and the composition of digestion products of olive oil was also established, where the mesophases mainly consisted of monoglycerides and protonated fatty acids.

In the early 2000s Borné et al. (2002) demonstrated the formation of specific mesophases during the digestion of oleic acid-based acylglycerol systems using a combination of CPLM, SAXS, and HPLC. Ternary phase diagrams were established to enable prediction of the mesophases formed at specific concentrations of monoolein and oleic acid/sodium oleate in water that can occur during lipolysis. It was established that decreasing the ratio of glyceryl monooleate to oleic acid will result in the phase transitions from V_2_(Im3m), to H_2_ phase, the I_2_(Fd3m) phase, and then the L_2_ phase (Borné et al., 2002). Meanwhile, increasing the ratio of glyceryl monooleate to sodium oleate will result in a phase transition of L_α_ to H_2_ phase.

**FIGURE 8:**
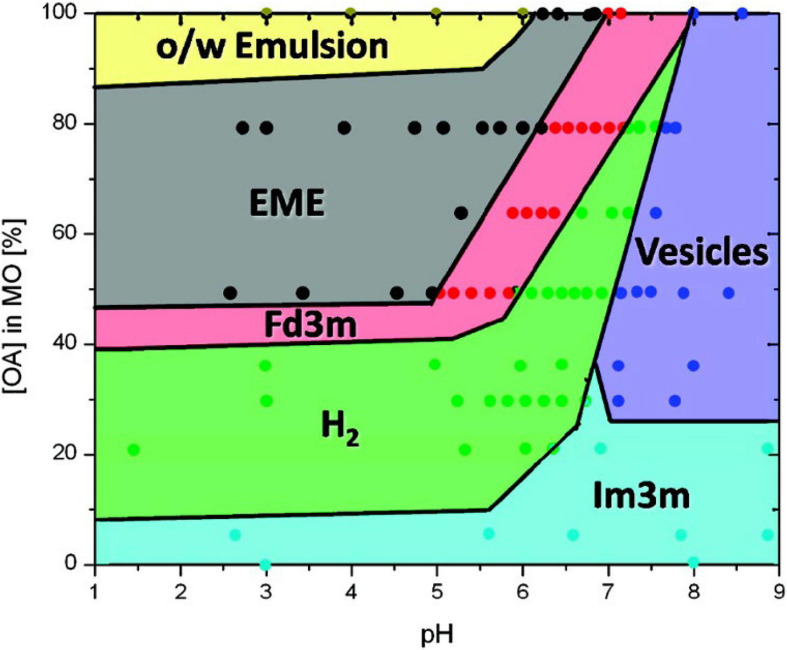
A phase behavior diagram of monoolein and oleic acid mixtures in PBS buffer with pH of 1–9 at 25°C, constructed by Salentinig et al. (2010a). Phases were determined *via* SAXS analysis of pre-made dispersions of the lipids. Adapted with permission from Salentinig et al. (2010a). Copyright (2010) American Chemical Society.

The prediction of the phases that can occur during the digestion of oleic-based acylglycerol systems was later expandedto encompass the changes in pH during *in vitro* digestion (Salentinig et al., 2010a). A myriad of structures was established from the combination of oleic acid and monoolein under different pH values, ranging from 1 to 9 (Figure 8). It was also discovered that the phase changes were reversible with pH. Additional cryo-TEM techniques were incorporated to observe the changes in structural morphology of the digesting lipids in conjunction with SAXS (Salentinig et al., 2010a).

Real-time structural evolution of digested triolein was then examined by using an *in vitro* digestion coupled to SAXS apparatus similar to that established by Fatouros et al. (2007) and Salentinig et al. (2010b). The complex LC structures that arise during the digestion of triolein were demonstrated to be in agreement with previous studies. The changes in mesophase observed during digestion were due to the CPP of the amphiphilic digestion products changing throughout the process.

## Mesophase structure Formation During Digestion in Pharmaceutical Applications

Mesophases possess both lipophilic domains and hydrophilic water channels which are capable of solubilizing both lipophilic and hydrophilic drugs, making them an ideal system for drug delivery (Nguyen et al., 2010, 2011a,b; Phan et al., 2011). Lipids have been utilized as a carrier for many poorly water-soluble drugs to improve their oral bioavailability (Pouton, 2000; Porter et al., 2007; Feeney et al., 2016). Lipid-based mesophases are also already in use in pharmaceutical applications, including liposomes (nano-sized lamellar phase) already on the market for the delivery of a range of drugs (Kaminskas et al., 2012; Negrini et al., 2015; Rabinovich et al., 2015), and for slow release injections (Haasen et al., 2017). The dispersed mesophases (comprising nano-sized mesophase particles) are often preferred over the bulk phase as the bulk phases are generally highly viscous (especially in the case of bicontinuous cubic V_2_ phase) and difficult to handle (Larsson, 1999). Traditional methods for generating dispersed phases require high levels of energy to break up the bulk aggregates to form stable sub-micron particles. A limitation to this approach is that heat is invariably generated which can degrade heat sensitive materials incorporated into the nanoparticles. Furthermore, this process is energy intensive and is therefore costly for manufacturing. The alternative “bottom up” approach is designed to allow the lipids to self-assemble into LC structures by dissolving lipids into hydrotropes, then diluting the mixture with an aqueous medium (Spicer et al., 2001). Precipitation that occurs within the cubic phase-water miscibility gap allows the formation of cubosomal dispersions (likewise for hexosomal dispersions). However, this process does not allow control of the particle size, which is often an essential requirement for pharmaceutical preparations used for intravenous (IV) administration or medical imaging applications.

**FIGURE 9:**
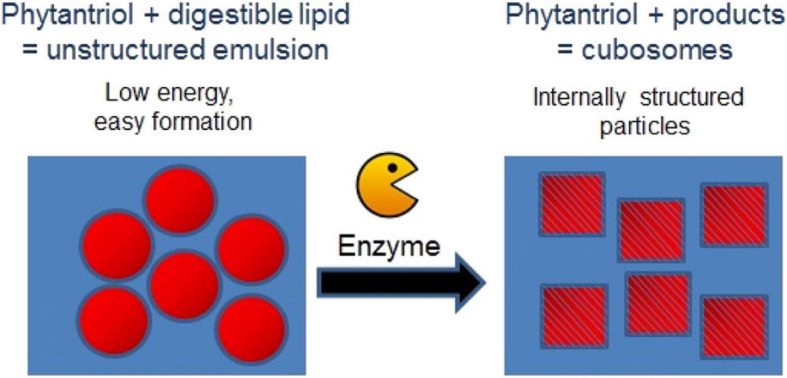
Schematic diagram demonstrating the enzymatic approach to form mesophases particles using a precursor formulation comprising phytantriol and a digestible lipid. Only low energy is required to generate the precursor formulation, in which the digestible lipids partition out of the formulation under digestion to enable the formation of the internally structured particles. Adapted with permission from Fong et al. (2014). Copyright (2014) American Chemical Society.

An alternative low energy approach to making mesophase dispersions using lipid digestion was recently studied. Precursor unstructured emulsion systems are prepared comprising a non-digestible and a digestible lipid (e.g., triglyceride) which after digestion by pancreatic lipase forms a dispersed mesophase system (Figure 9; Fong et al., 2014; Hong et al., 2015). For example, by using a precursor emulsion system composed of non-digestible phytantriol and the digestible short chain triglyceride, tributyrin, exposure to lipase resulted in digestion of the tributyrin, allowing the phytantriol to self-assemble into the bicontinuous cubic V_2_ phase (Lee et al., 2009; Nguyen et al., 2011b; Fong et al., 2014; Hong et al., 2015). As a proof of concept demonstration of the *in vivo* behavior of these enzymatically triggered delivery systems, oral administration of the easily formed phytantriol/tributyrin emulsion system containing a model hydrophobic drug cinnarizine, resulted in the same behavior as a formulation prepared using phytantriol alone (Hong et al., 2015; Pham et al., 2016). More recently, the enzymatic digestion of phospholipids to induce phase transformations has also been explored (Fong et al., 2019). The generation of diacylglycerol (DAG) from the action of phospholipase C was studied by NMR and time resolved SAXS was used to study the structural changes occurring during the digestion process. The type of phospholipid present had consequences for the disposition of the DAG digestion products with respect to the lipid bilayers with consequent impacts on self-assembled structures formed.

The type of mesophase formed during the digestion of lipid-based drug delivery systems is also an important factor to consider as different mesophases have not only been demonstrated to exhibit different rates of release but also vary in drug solubilizing capacity to affect the fate of drug absorption during digestion (Kossena et al., 2005; Phan et al., 2011; Yaghmur et al., 2012; Du et al., 2014). In cases where drug solubilization capacity is reduced upon dilution of the lipids in the GI tract, precipitation of the drug may occur and consequently cause a reduction in drug absorption (Khan et al., 2015a). The effect of phase behavior on drug absorption was demonstrated by Kossena et al. (2005) where drug absorption from the lamellar phase was reduced upon exposure to intestinal fluids compared to the V_2_ phase.

## Self-Assembly Behavior of Lipid-Based Food Digestion Products

In recent years, the self-assembly of lipids during the digestion of various food sources has gained interest. It has been hypothesized that the formation of the lipid mesophases is linked to the enhanced uptake of nutrients or to transport poorly water-soluble molecules through the aqueous intestinal fluids (Salentinig et al., 2013; Assenza and Mezzenga, 2019). Table 1 provides a summary of the different phase transitions that occur during the digestion of various food types. The mesophases described in Table 1 have been determined by conducting *in vitro* lipolysis coupled to SAXS.

**TABLE 1:** Summary of food type, the approximate lipid composition (SCT = short chain triglyceride, MCT = medium chain triglyceride, LCT = long chain triglyceride, FA = fatty acid, MG = monoglyceride), and the corresponding mesophase transitions that occur during digestion.

Food product	Percentage of fat (%) used in the starting emulsion	Approximate/average fatty acid composition (mol %)	Mesophase transitions during digestion*	References
Bovine milk (pasteurized and homogenized)	3.8	SCT: 0.4–12 MCT: 10–26 LCT: 74–90	Emulsion →L_α_ →L_α_ + I_2_(Fd3m) →L_α_ + H_2_ →L_α_ + H_2_ + V_2_(Im3m)	(Feng et al., 2004; Lindmark-Månsson, 2008; Salentinig et al., 2013; Clulow et al., 2018; Pham et al., 2020)

Human breast milk	2.20 ± 0.13 w/v	SCT: 0 MCT: 5–13 LCT: 87–95	Emulsion →L_α_ I_2_(Fd3m)	(Salentinig et al., 2015; Pham et al., 2020)

Goat milk (pasteurized and homogenized)	3.6	SCT: 2–5.7 MCT: 16–27 LCT: 63–78	Emulsion →L_α_ →L_α_ + I_2_(Fd3m) →L_α_ + H_2_ →L_α_ + V_2_(Im3m) →L_α_	(Prosser et al., 2010; Pham et al., 2020)

Soy juice	3.0	SCT: 0 MCT: 0–1 LCT: 99–100	Emulsion I_2_(Fd3m)	(Li et al., 2017; Pham et al., 2020)

Infant formula (three different brands)	(1) 3.8	(1) MCT: 3.45 ± 0.24 FA/4.03 ± 0.08 MG LCT: 95.57 ± 0.24 FA/95.97 ± 0.08 MG	(1) Emulsion →L_α_ →I_2_(Fd3m)	(Pham et al., 2020)
	(2) 3.8	(2) MCT: 13.51 ± 0.042 FA/8.15 ± 0.71 MG LCT: 95.57 ± 0.24 FA/95.97 ± 0.08 MG	(2) Emulsion →L_α_ →L_α_ + H_2_	
	(3) 3.6	(3) MCT: 4.90 ± 0 35 FA/3.89 ± 0.22 MG LCT: 93.81 ± 0.38 FA/96.11 ± 0.22	(3) Emulsion →L_α_	

Mayonnaise	7.15	SCT: 0 MCT: 0 LCT: 100 (Eastwood et al., 1963)	Emulsion →I_2_(Fd3m) + L_2_ →H_2_ →V_2_(Pn3m) →L_α_	(Salentinig et al., 2017)

Krill oil	5	SCT: 0 MCT: 0 LCT: 100	Emulsion →L_α_ →H_2_	(Codex Alimentarius Commission, 2017; Yaghmur et al., 2019)

*It is now likely that in many cases where the lamellar phase observed in digesting lipid systems that it is due to calcium soap formation rather than a putative L_*a*_ phase (Clulow et al., 2018).

### Mesophase Formation During the Digestion of Mammalian Milk

**FIGURE 10:**
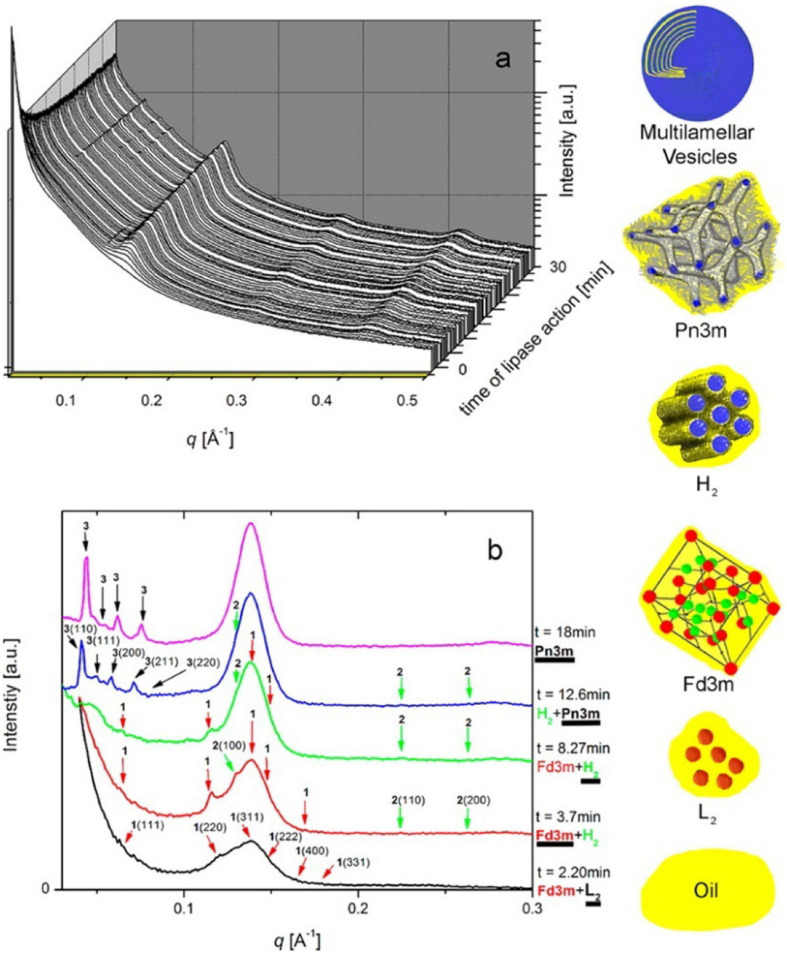
**(A)** A 3D waterfall plot of the scattering profiles obtained during the digestion of milk coupled to SAXS is presented. **(B)** Offset SAXS profiles of milk during digestion in the absence of bile salt is presented. The peaks corresponding to the various mesophases are indicated with colored arrows, with the corresponding schematic diagrams of the mesophases presented in order of appearance from bottom to top (excluding multilamellar vesicles). This figure was reproduced with permission from Salentinig et al. (2013). Copyright (2013) American Chemical Society.

By coupling SAXS with *in vitro* digestion models, it has been shown that the digestion of mammalian milks generates mesophase structures. Mammalian milks contain a myriad mixture of triglycerides and many other components that play a role in mesophase formation during digestion. Bovine milk contains approximately 3.5% fat, of which 98% are triglycerides, which undergoes digestion by lipases in the stomach and the upper section of the small intestine, the duodenum. There are over 400 individual different acyl chains that can make up the triglyceride molecules, leading to potentially thousands of different triglycerides (Lindmark-Månsson, 2008). Approximately 75–90% of the fatty acids found in the triglyceride core are long-chain, of which 30% are unsaturated (Lindmark-Månsson et al., 2003; Pham et al., 2020). Salentinig et al. (2013, 2015) reported the formation of mesophases during the digestion of milk using the pH-stat *in vitro* digestion model coupled to SAXS (Figure 10).

**FIGURE 11:**
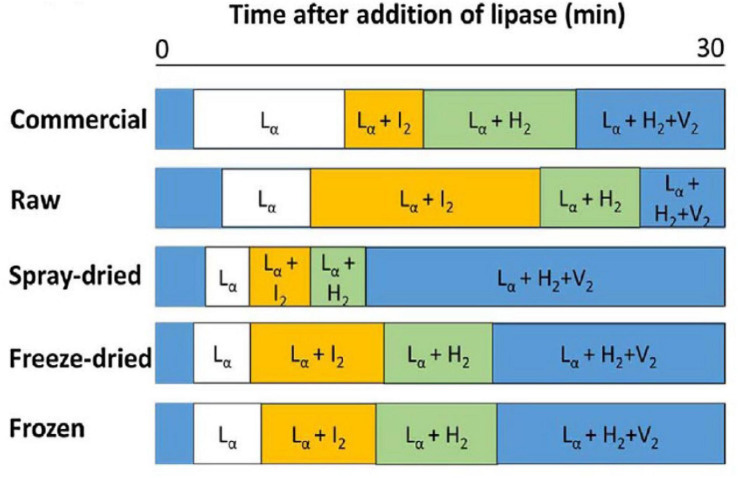
The typical phase transition during the digestion of bovine milk subjected to homogenization and pasteurization (commercial), raw, spray-dried, freeze-dried, and frozen milk reported and reproduced with permission from Clulow et al. (2018). L_α_ denotes lamellar phases formed by calcium soaps, I_2_ denotes the micellar cubic (Fd3m) phase, H_2_ denotes hexagonal phase, and V_2_ is the bicontinuous cubic (Im3m) phase.

The composition of lipids changes during digestion of milk due to their digestion, enabling various mesophases to form from the digestion products as a function of time (Pham et al., 2020). Typical phase transitions during the digestion of bovine milks that were subjected to various treatments are shown below in Figure 11 as illustrated by Clulow et al. (2018).

**FIGURE 12:**
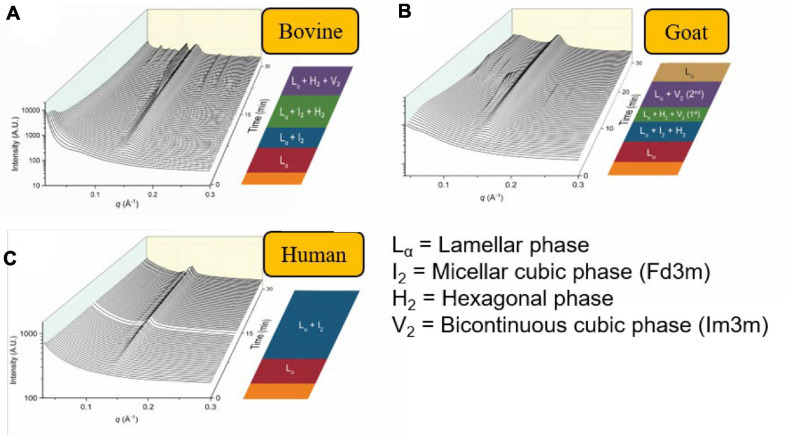
The formation of mesophases during the digestion of mammalian milk. Scattering profiles of **(A)** bovine milk, **(B)** goat milk, and **(C)** human milk during *in vitro* digestion coupled to SAXS are shown. Adapted and reproduced from Pham et al. (2020).

Similarly, other mammalian milks such as the human breast milk or goat milk have also been demonstrated to form non-lamellar mesophases during digestion (Salentinig et al., 2015; Pham et al., 2020). In particular, the scattering profiles of the digestion of goat milk resembled that of bovine milk. However, the persistent V_2_(Im3m) phase seen in bovine milk had disappeared toward the end of the digestion of goat milk. This was attributed to the greater abundance of short- to medium-chain triglycerides in goat milk in comparison to bovine milk (Li et al., 2017; Pham et al., 2020). The scattering profiles of bovine milk, goat milk, and human milk during *in vitro* digestion are shown below in Figure 12 (Pham et al., 2020). The differences in mesophase progression observed during the digestion of various types of mammalian milk is of interest as it may influence the absorption of lipophilic nutrients.

### Self-Assembly Behavior of Soy Lipids During Digestion

Emulsions prepared using vegetable oils such as canola oil, soybean oil or almond oil are popular as milk substitutes for those who prefer non-dairy beverages. Soy and other vegetable oils are rich in long-chain and unsaturated triglycerides, containing palmitic (C16:0), oleic (C18:1), linoleic (C18:2), and linolenic (omega 3, C18:3), and docosahexaenoic (omega 3, C22:6) fatty acids (Li et al., 2017; Pham et al., 2020). Foods enriched with omega 3 fatty acids have been shown to improve cardiometabolic profiles of people with underlying cardiovascular risks (Barbosa et al., 2017). Like mammalian milk, the triglycerides in soy milk are composed of different combinations of fatty acids on the glycerol backbone, although there are fewer combinations (Brockerhoff and Yurkowski, 1966; Innis, 2011). The digestion of certain brands of commercially available soy emulsions containing soy lipids has recently been demonstrated to form the I_2_(Fd3m) phase, similar to that of human breast milk although without the calcium soaps observed to form in digesting human breast milk. The authors suggested that the formation of the I_2_(Fd3m) phase was due to the abundance of linoleic acid, which was also the case for human breast milk (Pham et al., 2020). However, the significance of the individual mesophases on the absorption of lipophilic nutrients is yet to be determined.

### Self-Assembly Behavior of Infant Formula Lipids During Digestion

**FIGURE 13:**
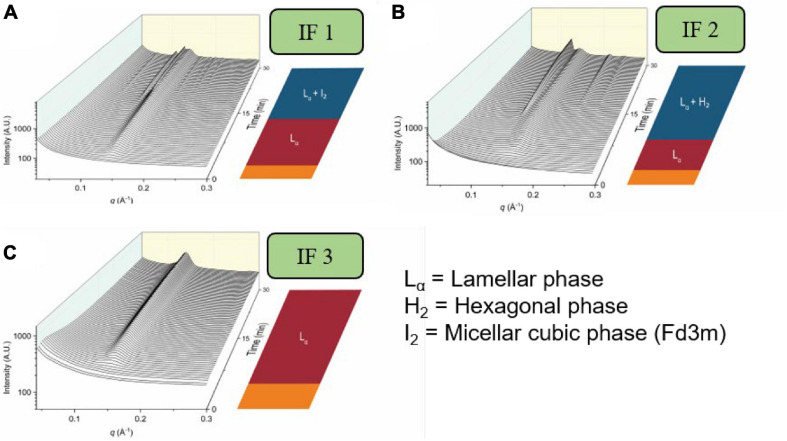
The formation of mesophases during the digestion of various brands of infant formula. Scattering profiles during the digestion of three brands of infant formula are adapted and reproduced from Pham et al. (2020). **(A)** represents the scattering profiles during the digestion of infant formula 1 (IF 1), **(B)** represents the scattering profiles during the digestion of infant formula 2 (IF 2), and **(C)** represents the scattering profiles during the digestion of infant formula 3 (IF 3).

Infant formula has been developed over the last century to try to mimic composition provided by the human breast milk and is certainly marketed as such. Infant formulae can contain fat sources from bovine milk, goat milk and vegetable oils depending on the brand and age of the child being provided the formula. Due to the differences in lipid composition, studies have shown that the self-assembly behavior of the lipids in various types of infant formulae exhibit different mesophases during digestion, shown in Figure 13 (Salim et al., 2019; Pham et al., 2020). The triglyceride content of emulsions attempting to mimic milk has recently been shown to be a key driver for the formation of mesophases commensurate with the milk of a particular species. The lipid self-assembly behavior of human breast milk during digestion was found to be replicated by relatively simple emulsions containing four–seven homotriglycerides mixed together, with a balance of long-chain saturated and unsaturated lipids (Clulow et al., 2020a). Similarly, the structural progression observed by digesting cow milk fat richer in medium and long-chain saturated lipids could be changed to that of digesting human breast milk by mixing it with canola oil, which comprises almost exclusively long-chain unsaturated lipids (Clulow et al., 2020b). These studies indicate the fine balance in lipid composition required to replicate the self-assembly behavior of human breast milk in infant formula products.

Milk is also increasingly recognized as a potential lipid-based drug delivery system due to its ability to dissolve poorly water-soluble drugs (Macheras and Reppas, 1986; Charkoftaki et al., 2010; Boyd et al., 2018; Binte Abu Bakar et al., 2019), which can be enhanced by the process of digestion (Boyd et al., 2018), however, its translation into pharmaceutical products is hindered by the variability in milk composition (Lindmark-Månsson et al., 2003; Lindmark-Månsson, 2008; Taylor and MacGibbon, 2011; Logan et al., 2014). Infant formula has therefore been suggested to play a role in drug delivery to circumvent issues with variability in milk powder. Infant formula is manufactured from a blend of fat sources and is available as a powdered form. The formula composition is tightly controlled to specifications in a manner similar to pharmaceutical excipients and the fat content can be controlled. Recent studies by Salim et al. (2019) showed improved solubilization of the drug clofazimine when digested with infant formula. In order to progress infant formula toward approval as a formal pharmaceutical excipient, more research is needed to understand its behavior during digestion including further studies on how the composition (lipids and other components) and overall fat content impact on structure and interaction with drug cargo.

### Examples of Liquid Crystalline Structure Formation in Other Food Products

Recently other food sources have been shown to generate non-lamellar mesophases during digestion. Mayonnaise which is a popular condiment that contains approximately 80% of triglycerides that are derived from vegetable oil. Unlike bovine milk, the triglycerides within mayonnaise are mainly composed of long-chain lipids, of which approximately 90% are unsaturated fatty acids (Eastwood et al., 1963). Interestingly, the mesophase progression of mayonnaise during digestion was found to be similar to that of bovine milk (except for the absence of the L_α_ phase), where the lipids of mayonnaise self-assemble into the I_2_(Fd3m)/L_2_ phase, followed the H_2_ phase, and finally the bicontinuous V_2_(Pn3m) phase (Salentinig et al., 2017). The absence of the L_α_ phase during the digestion of mayonnaise in comparison to bovine milk may be due to the lower proportion of palmitic acid in mayonnaise (∼20% palmitic acid in mayonnaise, ∼37% in bovine milk) which may otherwise contribute to lamellar diffraction peaks from calcium soaps formed during digestion (Ono et al., 1970; Pham et al., 2020). In addition to mayonnaise, krill oil was also found to form structured mesophases during *in vitro* digestion (Yaghmur et al., 2019). Krill oil is a supplement rich in omega-3 polyunsaturated fatty acids, mainly located in phospholipids (Bottino, 1975; Clarke, 1980). The digestion of krill oil resulted in the formation of L_α_ to H_2_ phase when studied using *in vitro* lipolysis coupled to SAXS (Yaghmur et al., 2019). The persistent H_2_ phase at the end of the digestion of krill oil may be linked to the abundance of long-chain lipid species in comparison to other systems like that of bovine milk. Therefore, the mesophase structure is more negatively curved relative to the bicontinuous V_2_ phase. Nonetheless, several examples of food products have been demonstrated to form complex mesophase systems during digestion which raises the question as to why this unique phenomenon occurs. These recent developments in understanding the dynamic structures formed during digestion may have implications on the transport and delivery of nutrients.

The recognition of the formation of mesophase structures in foods during digestion is becoming more widespread as new techniques are developed that can provide information on these phenomena. The broader question of “what is food?” is becoming an interesting one in this context – for example, mammalian milks have been shown to exhibit rich mesomorphism not seen in vegetable-sourced substitutes. This leads to a tentative sub-question of “what is milk?” – the broader question then of “what is food?” when comparing food from natural sources to formulated foods, may in future be in part answered through a classification based on the structural attributes of each during digestion. Differences in lipid composition at the level of fatty acid distributions are clearly important both for structure (Pham et al., 2020) and nutritional outcome (Li et al., 2010; Palmquist et al., 1986), so it remains an open question as to whether such a distinction eventuates with a link to broader health outcomes.

## Conclusion

The understanding of the formation of lipid-based mesophases during digestion has accelerated with the advent of time-resolved X-ray scattering capabilities. While the role of the formation of such structures in the digestion process is not yet completely understood, it is becoming clearer that it is a key feature of lipid digestion across a range of lipid-based materials from synthetic lipid systems through formulated food and pharmaceuticals to endogenous lipid materials such as breast milk. Establishing the link between formation of mesophases in lipid systems during digestion and nutritional outcomes is the next major step forward necessary to harness and utilize the phenomenon. It is also important from a food and pharmaceutical design standpoint as formulated systems have potential to either disrupt or enhance the formation of mesophases during digestion with consequences for nutrient and drug delivery.

## Author Contributions

AP: concept, researching literature, writing original draft, and reviewing and editing. AC: reviewing and editing. BB: concept, reviewing and editing, funding, and project administration. All authors contributed to the article and approved the submitted version.

## Conflict of Interest

The authors declare that the research was conducted in the absence of any commercial or financial relationships that could be construed as a potential conflict of interest.
